# Retrospective analysis of association between hepatopathy and serum DGGR lipase activity in dogs: a pilot study

**DOI:** 10.1177/10406387221106401

**Published:** 2022-06-27

**Authors:** James M. Thomson, Tim L. Williams

**Affiliations:** Department of Veterinary Medicine, University of Cambridge, Cambridge, UK

**Keywords:** 1,2-o-dilaurl-rac-glycero-3-glutaric acid-(6’-methylresorufin) ester, DGGR lipase, dogs, hepatitis, hepatopathy, liver, pancreas, pancreatitis

## Abstract

1,2-o-dilauryl-rac-glycero-3-glutaric acid-(6′-methylresorufin) ester (DGGR) lipase assays are used to measure lipase activity in the diagnosis of pancreatitis. The effect of hepatic lipases released from damaged hepatocytes on serum DGGR lipase activity has not been reported, to our knowledge. We identified dogs with histologically confirmed liver lesions and concurrent unremarkable pancreatic histology, and dogs with no histologic evidence of hepatic or pancreatic disease. Dogs with relevant comorbidities were excluded. The *hepatopathy group* (*n* = 7) included 4 dogs with inflammatory hepatopathies, 2 with hepatic neoplasia, and 1 with unspecified (non-inflammatory, non-neoplastic) hepatopathy. The *control group* (*n* = 5) included one dog each with enteritis, subcutaneous hemangiosarcoma, hydrocephalus, myelomalacia, and tetanus. A Mann–Whitney U test compared selected biochemical parameters including serum DGGR lipase, alkaline phosphatase, alanine aminotransferase, and amylase activities, with statistical significance defined as *p* *≤* 0.05. Data are presented as median and range. Serum DGGR lipase activity (RI: <44 IU/L) was not different between the hepatopathy (52 IU/L; range: 27–85 IU/L) and control (37 IU/L, 25–105 IU/L; *p* = 0.947) groups. Serum amylase activity (RI: 256–1,610 IU/L) was significantly higher in the hepatopathy group (830 IU/L; 711–1,210 IU/L) than the control group (541 IU/L, 336–695 IU/L; *p* = 0.028). No association or correlation between serum DGGR lipase activity and hepatic lesions (based on histologic or biochemical findings) was identified, suggesting that clinically relevant changes in serum DGGR lipase activity may not be expected secondary to hepatopathy alone.

Serum lipase activity is usually measured clinically as a marker of pancreatic disease. However, there are several other canine lipases, including hepatic lipase, lipoprotein lipase, and gastric lipase, which may also be detected using commercial lipase assays, hence affecting the specificity of serum lipase activity as a marker of pancreatic disease.^13^

1,2-o-dilauryl-rac-glycero-3-glutaric acid-(6’-methylresorufin) ester (DGGR) is a substrate used to measure lipase activity, with DGGR lipase assays now utilized commonly for the diagnosis of canine pancreatitis. DGGR lipase assays are reported to be more specific than traditional 1,2-diglyceride assays for the detection of pancreatic lipases,^3^ and they are also reported to be as accurate as Spec cPL for the diagnosis of pancreatitis in dogs.^2,6^ DGGR lipase activity may also be affected by other non-pancreatic conditions, such as renal disease (although the association between renal disease and DGGR lipase activity remains controversial).^8,12^ Hyperlipasemia (determined using the older 1,2-diglyceride–based assay) has also been associated with hepatic neoplasia.^9^ Lack of specificity of the DGGR lipase assay for the detection of pancreatic lipase has been suggested, given that heparin administration, which will stimulate release of hepatic and lipoprotein lipases, caused a small (<10 IU/L), but statistically significant, increase in serum DGGR lipase activity in healthy dogs and cats.^7^

To our knowledge, the effect of histologically confirmed hepatopathy alone on serum DGGR lipase activity (presumptively through release of hepatic lipases) has not been reported. Anecdotally, we have observed mild elevations in serum lipase activity in dogs with elevated liver enzyme activity (alanine aminotransferase [ALT], alkaline phosphatase [ALP]) but without a clinical suspicion of pancreatitis, which could reflect release of hepatic lipases in dogs with hepatopathy. Therefore, our aim was to evaluate serum DGGR lipase activity in dogs with histologically confirmed liver lesions but with histologically normal pancreases, and to compare these results to serum DGGR lipase activities in dogs with histologically normal liver and pancreas (control dogs). We hypothesized that serum DGGR lipase activity would be greater in dogs with histologically confirmed liver lesions than in control dogs.

We searched postmortem database records of the Queen’s Veterinary School Hospital, University of Cambridge, United Kingdom between 2015 and 2020 for dogs with histologic evidence of hepatopathy and unremarkable pancreatic gross examination and histology. Dogs were considered to have hepatopathy if a compatible microscopic diagnosis and description (e.g., hepatic necrosis, hyperplastic hepatocytes, inflammation, neoplasia) was reported by a board-certified histopathologist or a resident working under their supervision. The postmortem database was also searched for dogs without gross or histologic evidence of hepatopathy or pancreatic lesions to serve as a control group. In control cases, the pancreas had been considered grossly normal, and a histologic assessment had been performed on a single, randomly selected section of pancreas. Clinical data collected included signalment, clinical presentation, clinical diagnosis, and cause of death. Dogs without serum biochemistry results (including DGGR lipase activity) available, or that had comorbidities or exogenous factors known to affect serum lipase activity, including heart disease,^4^ intervertebral disc disease,^10^ azotemia,^8^ or steroid administration,^4,8^ were excluded. For dogs with repeated biochemistry results available, the results closest to the date of postmortem examination were included. Dogs in the hepatopathy group were grouped according to etiology of the hepatopathy: inflammatory, neoplastic, or non-inflammatory and non-neoplastic.

Serum lipase activity was measured using a canine DGGR lipase assay (Lipase assay DGGR; Randox), validated previously for use in dogs.^11^ Serum amylase activity was measured using a commercial assay (α-amylase assay; Beckman Coulter). Serum ALT and ALP were measured using commercial assays (Beckman Coulter). All assays were performed using an Olympus AU480 analyzer (Beckman Coulter), which was controlled daily using 2 levels of commercial quality control material (Omnicore 1+2; Thermo Scientific). Data are presented as median and range.

Selected biochemical analytes were compared between the hepatopathy and control groups using the Mann–Whitney U test. Correlations were evaluated using the Spearman correlation coefficient. Statistical significance was defined as *p* ≤ 0.05.

Searching the keywords associated with hepatopathy, including hepatopathy, hepatitis, hepatic necrosis, and hepatic neoplasia, returned 189 matching results. Of those, only 7 had biochemistry blood results available and met the histologic and exclusion criteria. To comprise the control group, liver and pancreas within normal limits was searched, which returned 390 results. Of those, 13 had biochemistry results available and met the histologic criteria. Three were excluded because of azotemia, 3 because of heart disease, and 2 because they had received corticosteroids. The final hepatopathy group comprised 5 male dogs and 2 female dogs with a median age of 10.7 y (range: 8–11.4 y; Table 1). The breed distribution was 2 crossbreed dogs, and 1 each of Airedale Terrier, Bichon Frise, Cocker Spaniel, Greyhound, and Miniature Schnauzer. The final control group comprised 3 male dogs and 2 female dogs with a median age of 3.3 y (0.6–8.2 y; Table 1). The breed distribution was 1 each of Basset Hound, German Shepherd, Golden Retriever, Labrador Retriever, and Pug. The etiology of disease in the hepatopathy group included inflammatory (*n* = 4), neoplastic (*n* = 2), and non-inflammatory and non-neoplastic (*n* = 1; Table 2).

**Table 1.** Selected clinical and clinicopathologic data for dogs included in our study of the association between hepatopathy and DGGR lipase activity.

Variable	All dogs	Hepatopathy group	Control group	Significance	Laboratory RI
No. of dogs	12	7	5	NA	NA
Female	4	2	2	NA	NA
Male	8	5	3	NA	NA
Age, y	8.3 (0.6–11.4)	10.7 (8–11.4)	3.3 (0.6–8.2)	**0.02**	NA
Serum ALT activity, IU/L		258 (33–790)	38 (26–68)	**0.048**	14–67
Serum ALP activity, IU/L		737 (184–8,260)	76 (24–135)	**0.003**	26–107

Laboratory RIs were derived from a group of 20 healthy dogs. Data are presented as median (range). The Mann–Whitney U test was used to compare data between groups. Boldface indicates variables for which there was a significant difference between the hepatopathy and control groups.

ALP = alkaline phosphatase; ALT = alanine aminotransferase; NA = not applicable.

**Table 2.** Histologic and gross pathology diagnoses associated with each category within the hepatopathy and control groups.

Group	Histologic diagnosis	No. of animals
Hepatopathy	Inflammatory	
	Chronic hepatitis	2
	Necrotizing hepatitis	2
	Neoplastic	
	Hepatic adenoma	1
	Hepatic carcinoma	1
	Non-neoplastic, non-inflammatory	
	Unspecified hepatopathies	1
Control	Gastrointestinal	
	Enteritis	1
	Neoplastic	
	Subcutaneous hemangiosarcoma	1
	Congenital	
	Hydrocephalus	1
	Neurologic	
	Myelomalacia	1
	Tetanus	1

The number of days between date of blood sample and postmortem examination was 7 (0–162) d; 7 (0–107) d in the hepatopathy group and 7 (2–162) d in the control group. Serum ALT and ALP activities were significantly greater in the hepatopathy group compared to the control group, as expected (*p* = 0.048, *p* = 0.0025, respectively; Table 1). There were no significant differences in serum DGGR lipase activity between the hepatopathy and control groups (52 [11–98] IU/L vs. 37 [14–109] IU/L, respectively; *p* = 0.95; Fig. 1A). Serum amylase activity was significantly higher in the hepatopathy group than the control group (1,010 [415–1,390] IU/L vs. 541 [351–807] IU/L; *p* = 0.03; Fig. 1B). There was no clear pattern in serum amylase or lipase activities between different groups based on the etiology of the hepatopathy (Fig. 1A, 1B); however, the groups were too small to permit statistical analysis. Serum DGGR lipase activity was not significantly correlated with serum ALT activity (*r_s_* = −0.11, *n* = 12; *p* = 0.72) or serum ALP activity (*r_s_* = 0.028, *n* = 12; *p* = 0.93). Serum amylase activity was moderately, positively correlated with serum ALP activity (*r_s_* = 0.59, *n* = 12; *p* = 0.045) but was not significantly correlated with serum ALT activity (*r_s_* = 0.44, *n* = 12; *p* = 0.15).

**Figure 1.**
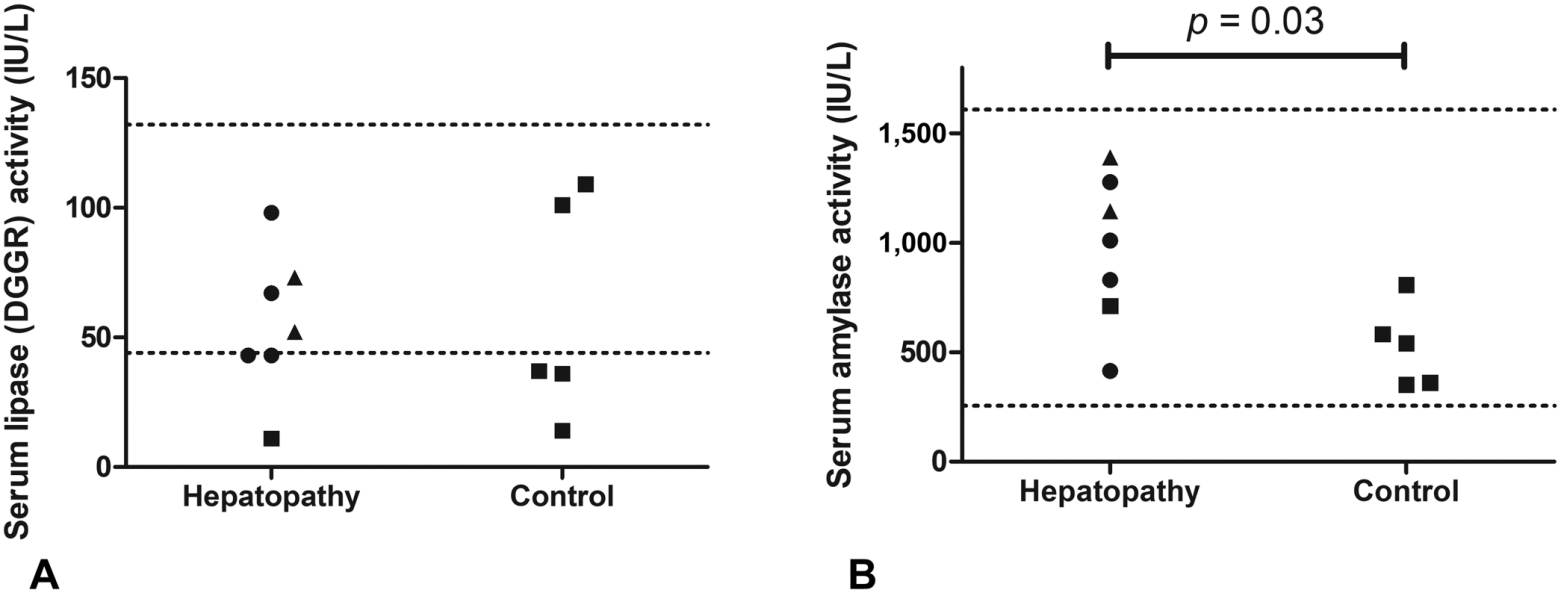
Scatter plots of serum DGGR lipase and amylase activities in the hepatopathy and control groups of dogs. In the hepatopathy group, circles represent data for dogs with an inflammatory condition; triangles represent data for dogs with neoplasia; squares represent data for the dog with a non-neoplastic and non-inflammatory condition. **A.** Serum DGGR lipase activities in the hepatopathy and control groups. The lower dotted line is the upper limit of the laboratory RI; the upper dotted line is 3× the upper limit of the RI, used to denote significantly increased activity.^8^ The Mann–Whitney U test was used to compare data between groups. There was no significant difference in serum DGGR lipase activity between the groups (*p* = 1.00). **B.** Serum amylase activities in the hepatopathy and control groups. The dotted lines are the upper and lower laboratory RI limits. The Mann–Whitney U test was used to compare data between groups. Serum amylase activities were significantly greater in the hepatopathy group compared to the control group (*p* = 0.03).

DGGR lipase activity has been found to increase significantly (albeit <10 IU/L) following administration of heparin at 1 of 5 times assessed,^7^ with a small median increase of 4.3 U/L 10 min after IV heparin administration, which, given that heparin is known to cause a release of hepatic and lipoprotein lipases, suggested that either hepatic or lipoprotein lipases can also hydrolyze the DGGR substrate. Our results suggest that hepatic lipases released in hepatopathy do not increase serum DGGR lipase activities to a clinically significant extent, given that no significant elevation (>3× upper RI) was observed in the hepatopathy group, and DGGR lipase was not significantly increased in this group compared to a control group of dogs with histologically normal pancreases. One possible explanation for the discordance between our data and the aforementioned study^7^ is that the elevation in DGGR lipase activity seen following administration of heparin is the result of the effects of lipoprotein lipase rather than hepatic lipases. Another explanation could be that serum hepatic lipase activities are only increased in certain types of hepatopathy, given that serum lipase activities (determined using the 1,2-diglyceride assay) were increased in dogs with hepatic neoplasia, in another study.^9^

Although there were only 2 dogs with hepatic neoplasia, and numbers of other types of hepatopathy were small in our study, we noted no obvious association between disease etiology and serum DGGR lipase activity, and no dogs had DGGR lipase activity >3× the upper limit of the RI. However, our small group sizes did not permit meaningful statistical comparison between the groups, hence further studies using larger numbers of dogs with different etiologies of hepatopathy would be required to confirm these findings. In addition, 2 dogs in the control population had mildly elevated serum DGGR activity (above the upper limit of the laboratory RI, but <3× the upper limit of the RI, which is the value often used clinically to signify significant elevation^8^); such elevation may 1) reflect hydrolysis of other lipases (e.g., lipoprotein lipase, gastric lipase) by the DGGR assay, 2) could reflect the possibility that our RI is too narrow, 3) could reflect normal biological variability, or 4) could be explained by the presence of pancreatic lesions that were not identified histologically. However, no dogs in the hepatopathy group had serum DGGR lipase activities >3× the upper limit of the RI.

Unexpectedly, we observed a significant increase in serum amylase activity in dogs with hepatopathy compared to the control group, and serum amylase activity was correlated with serum ALP activity, although serum amylase was within RIs in all cases. The 3 dogs in the hepatopathy group with the highest serum amylase activities all had mildly elevated serum DGGR lipase activities (range: 52–98 IU/L); therefore, it is possible that some of these dogs may have had mild pancreatitis that was not detected histologically. Alternatively, amylase activity and mRNA for amylase are present in canine liver,^13^ and humans with liver disease associated with functional impairment have decreased serum amylase activities^1^; therefore, hepatic-derived amylase could contribute to serum amylase concentrations in dogs, albeit to a small extent. Biliary amylase activity is also correlated positively with the degree of biliary hyperplasia in humans with choledochal cysts,^5^ suggesting that amylase might originate from biliary cells and be released secondary to biliary cell injury.

The retrospective nature of our study was a limitation because the time between blood sampling and postmortem examination could not be standardized. Although the median time between serum biochemistry analysis and the postmortem examination was 7 d, some samples predated postmortem examination by up to 162 d, meaning that the biochemistry results may not be correlated with the postmortem findings. However, given that animals in the hepatopathy group had been blood sampled because they had clinical signs compatible with hepatopathy, it seems likely that a hepatopathy did exist at the time of blood sampling, and therefore, if serum lipase activity was increased in these patients secondary to hepatopathy, then it should still be apparent in our data.

Using histopathology to define pancreatic lesions unfortunately meant only a small number of animals could be included in our study; however, this did allow us to minimize the risk of inclusion of cases with concurrent pancreatitis, which otherwise would confound our study findings. The pancreas was examined grossly, and histologic assessment was performed on a single, randomly selected sample. The final number of animals that complied with the inclusion and exclusion criteria was 12, which is very small and thus a large limiting factor for our study; therefore, our results only represent a pilot analysis, and additional studies using larger numbers of dogs should be performed. It could be speculated that our study is statistically underpowered to detect significant differences; however, post hoc power analysis suggests that, at a 5% level of significance, only 5 dogs would be needed in each group to have 80% power to detect a 3-fold (and thus likely clinically relevant) increase in the serum DGGR lipase activity in the hepatopathy group versus control dogs. Therefore, our study should be appropriately powered to detect such differences if they existed, although additional studies in larger cohorts of dogs would be warranted to confirm our findings and to explore if different subcategories of hepatopathy (e.g., hepatic neoplasia) are associated with hyperlipasemia when the DGGR lipase assay is used.
